# A Case of Hepatic Abscess, in Which Biliary Calculi Were Discharged at the Side, after an Operation Resulting Favorably

**Published:** 1841-05

**Authors:** William Dawson Dorris

**Affiliations:** Nashville, Tennessee


Art. IV.—*A Case of Hepatic Abscess, in which Biliary Calculi were discharged at the side, after an operation, resulting favorably.* By William Dawson Dorris, M. D., of Nashville, Tennessee.

The following case was discharged from my care a few months since, and the fortunate issue of it under the most unpromising circumstances, together with the unusual number of calculi discharged after an operation, I believe, not often resorted to, induce me to hope that it will not be found uninteresting to the readers of the Journal. The subject of it was a lady, Mrs. E. Y., æt. about 31 years, corpulent, and of the phlegmatic temperament. In February, 1838, I was first requested to visit her, and found her laboring under prolapsus uteri, attended with diarrhœa. Her menstrual function was also deranged, and at each monthly period the right ovary was so enlarged as to be distinctly felt on examination. In April she labored under the symptoms of internal polypus of the uterus, with uterine hemorrhage at the menstrual periods. About this time she had a copious discharge of a dark colored fluid, per vaginam, which gave her great relief, and the enlarged ovary ceased to be perceptible; her catamenia also returned and her health appeared to be much improved. About this time, however, she began to complain of pain in the region of the liver, for which I prescribed mercurial cathartics, Dover's powder, and warm fomentations. Her bowels were torpid, and required habitually to be moved by cathartics. In September she was attacked with bilious remittent fever, from which she was recovered by the usual treatment in two weeks.

November 1st. the patient was relieved of a polypus as large as a duck egg, and with it of the hectic flushings, the uterine hemorrhage, and all the other unpleasant symptoms which denoted its presence. But symptoms of ovarian disease still existed, and in March I found her suffering again with prolapsus uteri, and torpor of the liver and bowels. The uterus was restored to its place, a saline draught administered to move her bowels, and morphine given for the severe pain.

Under the treatment which was modified from day to day the sufferings of the patient were mitigated, but on the 13th of April I found the liver and right ovary much enlarged Ordered the skin over those parts to be rubbed with ung. hydrarg.

16th. Gave jalap 15 grains and rhubarb 5 grains.

18th. Found medicine had acted well, my patient sitting up and feeling much better.

22d. Enlargement of her side increasing rapidly, and for the first time during her illness, she is losing flesh. Complains of great burning in the right side, and is slightly salivated by the mercurial inunctions.

25th. Patient has been more comfortable for several days, and the indications of abscess of the liver are such that I proposed an operation; but as many physicians had been consulted on the case and had not agreed as to the nature of the affection, the lady objected.

May 1st. I had the pleasure of meeting Dr. F. Robertson in consultation. The tumor in the region of the liver was now eight inches in width, and twelve inches long. It was easy to perceive the interstice between that organ and the ovary, though their unnatural growth had brought them into contact. Blisters were applied over the tumor, with blue pill, sup. tart. potas. aloes and rhubarb to keep the bowels soluble. Fomentations to be continued.

2d. Blister drew well; purgatives acted freely, and the bile discharged produced great irritation in the rectum. Blue pill with hyosciamus to be continued.

5th. Blue pill discontinued, ptyalism having come on. Patient is discharging a soot-colored fluid through the vagina, and the enlargement of the ovary is visibly on the decline, while that of the liver is increasing.

9th. Patient very restless; has had shiverings, and syncope; pain in the right side, back and head, excruciating.

12th. A discharge from the bowels, of most offensive odor, appeared, and of black, yellow, green and white colors mixed. This matter continued to pass off from her bowels for a few days, and on the 15th she began to vomit a fluid of the same description.

16th. Patient better—hyosciam. pills to be continued, with mild purgatives.

21st. The enlargement of her side amounts to a deformity. She has passed five biliary calculi per anum.

22d. Visited her in company with Dr. Robertson, who advises her to submit to having the tumor punctured.

23d. Operated, by making a transverse incision two inches and a half in length through the integuments, and about two inches through the muscles down to the liver, which was found attached to the parietes of the abdomen. About a quart of such matter as she had discharged by the bowels flowed out, when faintness came on and made it necessary to check the flow for the time. Up to the 31st., the abscess continued to discharge freely at each dressing; the patient is so far improved as to be able to sit up.

June 6th. The incision being kept open, I extracted five biliary calculi of the size and shape of a beech nut, some of which when recently removed weighed twelve grains.

8th. Three biliary calculi removed at this dressing. Patient recovering.

11th. I extracted at this visit eighteen calculi, fourteen of which had a common, very thin, membranous envelope.

August 1st. Patient improves, using pil. hydrarg. and hyosciam. pro re nata. Up to this date one hundred and one gall stones have been extracted through the incision.

27th. Patient is able to attend to her household affairs. From this period until the 18th of December, ten more calculi were extracted, when the orifice began to close.. Thus, in all, one hundred and eleven calculi were taken away by instruments, and five were discharged per vias naturales. The health of the lady continued steadily to improve after the opening of the abscess, and it is better in every particular than she has enjoyed for many years.

I have not pretended, in the history of this case, to give all the minutiæ of treatment pursued during its progress, as there was nothing peculiar in it, and as the result did not appear to be brought about by the medicinal agents employed. It is a case beautifully illustrating the *vis midicatrix naturæ*—that conservative power by which offending matters in a deep seated and vital organ are directed to the surface of the body and there discharged.

*March* 17, 1841.

